# Structural and Optical Modifications in the BaO-ZnO-LiF-B_2_O_3_-Yb_2_O_3_ Glass System after *γ*-Irradiation

**DOI:** 10.3390/ma14226955

**Published:** 2021-11-17

**Authors:** Nimitha S. Prabhu, Hiriyur Mallaiah Somashekarappa, M. I. Sayyed, Hamid Osman, Sultan Alamri, Mayeen Uddin Khandaker, Sudha D. Kamath

**Affiliations:** 1 Department of Physics, Manipal Institute of Technology, Manipal Academy of Higher Education, Manipal 576104, India; nimprabhu14@gmail.com; 2 Centre for Application of Radioisotopes and Radiation Technology (CARRT), Mangalore University, Mangalagangothri 574199, India; carrtmu@gmail.com; 3 Department of Physics, Faculty of Science, Isra University, Amman 11622, Jordan; dr.mabualssayed@gmail.com; 4 Department of Nuclear Medicine Research, Institute for Research and Medical, Consultations (IRMC), Imam Abdulrahman Bin Faisal University (IAU), P.O. Box 1982, Dammam 31441, Saudi Arabia; 5 Department of Radiological Sciences, College of Applied Medical Sciences, Taif University, Taif 21944, Saudi Arabia; ha.osman@tu.edu.sa (H.O.); s.alamri@tu.edu.sa (S.A.); 6 Center for Applied Physics and Radiation Technologies, School of Engineering and Technology, Sunway University, Subang Jaya 47500, Malaysia; mayeenk@sunway.edu.my

**Keywords:** irradiation, glass, optical properties, structural properties

## Abstract

A Yb^3^^+^-doped borate glass system was examined for the structural and optical modifications after γ-irradiation. Among the studied 10BaO-20ZnO-20LiF-(50-*x*)B_2_O_3_-*x*Yb_2_O_3_ (*x* = 0.1, 0.5, 0.7, and 1.0 mol%) glasses, the 10BaO-20ZnO-20LiF-49.9B_2_O_3_-0.1Yb_2_O_3_ glass showed the highest thermoluminescence intensity, trap density, and trap depth. The glass was irradiated with the optimum γ-dose of 1 kGy towards the analysis of radiation-induced defects. The amorphous nature was preserved before and after irradiation. The glass density slightly increased after irradiation. The structural rearrangement was evident from the Fourier transform infrared spectroscopy by the appearance and disappearance of some bonds after γ-irradiation. The transformation of [BO_4_] units into [BO_3_] units and non-bridging oxygens was deduced. The color of the glass darkened after irradiation and the optical absorption intensity enhanced between 250 and 700 nm. The optical bandgap reduced and Urbach energy increased upon γ-dose exposure. The electron spin resonance of the irradiated glass exhibited two signals at g = 2.0167 and g = 1.9938, corresponding to the non-bridging oxygen hole center and Boron *E’*-center, respectively.

## 1. Introduction

The interest in researching the radiation effects on glasses, which began in the 1950s, has been growing enormously since then [1,2,3,4,5]. The glass properties are thoroughly linked to the inter-atomic forces and potentials in the lattice [6]. It is accepted that, when a glass is subjected to ionizing γ-radiation, it acquires some ‘radiation-induced defects’, which alter its network [3]. The interaction of a glass with the incident γ-rays is linked to the rate of creation and buildup of such radiation-induced defects during the course of irradiation [7]. These defects may arise because of atomic displacement by momentum and energy transfer, charge trapping, ionization, and/or radiolytic effects. The interaction processes in a material occurring due to γ-rays are mostly through the photoelectric effect, Compton scattering, and pair production. At ≤10 kGy levels, electron–hole pairs are formed in the glass, some of which dissociate and are trapped at sites intrinsically present [2]. The radiation interaction may include alterations in the valency of the lattice or impurity ions [8]. Generally, the term ‘defect centers’ is ascribed to the electrons/holes trapped at different lattice imperfections in the glass network. Such defects mainly affect the structural and optical characteristics of the glass [9]. The nature and the concentration of the defect centers vary with the dose, preparation technique of the material, and its composition. 

The fundamental methods for defect analysis in glasses have been density [3,7], optical absorption spectroscopy [8,9,10,11], Fourier transform infrared (FTIR) spectroscopy [9,11], and electron spin resonance (ESR) [1,2,12]. Advances in science have expanded the experimental approaches to some luminescence techniques like thermoluminescence (TL) [13,14,15,16], radio-thermoluminescence (RTL) [10], and photoluminescence (PL) [17]. Every characterization technique has its significance in the analysis of resultant radiation-induced defects in the glass. Measurement of glass density before and after irradiation reveals the expansion or contraction that occurred in the glass [3]. Irradiation can modify the glass optical properties by the presence of new absorption bands in the spectrum [8]. The optical absorption and emission of the glass after irradiation give an idea of the extent of photo-darkening in the ultraviolet (UV), visible (Vis), and infrared (IR) regions [18]. Based on this, the radiation hardness of the glass can be reckoned for the development of optical fiber, and photonic systems in an environment like space [18]. From the optical absorption spectroscopy, the absorption edge helps study the band structure, optically induced transitions, and optical bandgap [6]. Any modification in the lattice structure due to irradiation can be detected by FTIR spectroscopy [6]. The FTIR spectroscopy helps in distinguishing the bonds forming the glass network, and the consequences of irradiation on them [7,9,11]. For the identification of paramagnetic defect centers, i.e., the centers having unpaired electrons, the ESR is a conventionally used technique [1,2]. The ESR also provides information on the nature of defect sites in the glass structure from their characteristic g-values [1,19]. The TL glow curve offers information on the activation energy, lifetime, and wavelength of the defect centers [13,15,20]. Altogether, the examination of the structural and optical features of the glass pre- and post-irradiation is useful for studying the intrinsic glass structure and the changes resulting from the interaction with radiation. From a practical point of view, the radiation-induced changes are essential for knowing the evolution of the glass under long-term irradiation while it is being used for any application in a radiation environment.

The borate host glass can be formed easily at relatively lower melting temperatures [21]. However, as the pure borate glass gives out a feeble TL glow [22], it cannot be used as it is for dosimetry. The addition of modifiers is required to solve this problem. LiF as a modifier in the present work was added in 20 mol% owing to its virtue of being used in TL readers as a ‘routine dosimetry system’ for γ-doses of 10^−4^–10^3^ Gy [23]. Moreover, in (Li_2_O)_30-x_(Li_2_F_2_)_x_(B_2_O_3_)_70_ glasses [22], replacing Li_2_O by LiF (x = 10) caused the appearance of an additional TL peak around 415 K, the intensity of which had increased with LiF doping. The high-temperature peak was favorable towards the effective application of the glass in dosimetry. The addition of ZnO to the borate glass can improve its thermal stability and mechanical rigidity [24]. To enhance the glass density and provide resistance against ionizing radiation, a heavy element oxide like BaO is introduced in the glass composition [25]. In the ZnO-Na_2_O-H_3_BO_3_-BaO glass system [25], not only the number of bonds per unit volume increased with BaO addition, but also the radiation attenuation characteristics improved and the glass durability was maintained until 15 mol% BaO content. The rare-earth ions can influence the mechanisms responsible for the formation of the radiation-induced color centers [10] and even change the TL features of the host [16]. The ytterbium rare-earth ion is commonly used in optical fiber and laser applications [26,27]. Not just limited to photonic applications, the work on the inclusion of ytterbium oxide in the sodium tetra-borate glass had led to a shift of the RTL peak to higher temperatures and suppression of the low-temperature peak at 90 °C towards dosimetry application [10]. This had motivated us to take up the study on TL dosimetry application of Yb^3+^-doped glass. Previously [13], we had investigated the 10BaO-20ZnO-20LiF-(50-*x*)B_2_O_3_-*x*Yb_2_O_3_ (*x* = 0.1, 0.5, 0.7, and 1.0 mol%) glass system for TL dosimetry and obtained the highest integrated TL intensity at the 0.1 mol% doping level. The glass seemed to be suitable for food irradiation application in the dose range of 0.25 to 1 kGy, which was comparable to other rare-earth doped glasses like PbO–Al_2_O_3_–SiO_2_:Nd^3+^ [14], Dy^3+^-doped lithium borate [16], and BaO–B_2_O_3_–P_2_O_5_–Al_2_O_3_–Tb_2_O_3_ [15]. The glass showed quite high sensitivity, a threshold dose of 166 ± 3 Gy, and an effective atomic number of 9.7. In the present work, we continue the study of this glass system by analyzing the radiation-induced defects through optical and structural properties.

## 2. Materials and Methods

The conventional melt-quench method was used to synthesize the 10BaO-20ZnO-20LiF-(50-*x*)B_2_O_3_-*x*Yb_2_O_3_ (*x* = 0.1, 0.5, 0.7, and 1.0 mol%) glasses, as mentioned in our previous work [13]. In brief, all the mentioned chemicals were grinded, and the mix was heated inside an alumina crucible at 1100 °C, which was kept inside a furnace for 1 h. The melt was quenched on a preheated stainless-steel mold at 350 °C. Annealing was sustained at the same condition for 3 h. The respective ZLBBY0.1, ZLBBY0.5, ZLBBY0.7, and ZLBBY1 glasses polished to 3 mm were characterized. 

Characterization methods:

- Irradiation:

Gamma Chamber: GC-5000, BRIT, Mumbai, India.

Source: ^60^Co.

Dose rate: 3 kGy/h.

b. TL:

Sample specification: glass powder of weight 0.15 g.

Instrument: TLD reader 1009I, Nucleonix systems Pvt. Ltd., Hyderabad, India.

Heating rate: 2.85 K/s.

For glow curve deconvolution: Levenberg–Marquardt algorithm composed in Wolfram Mathematica software 12.1.

c. Density:

Instrument: Contech density balance, Contech Instruments Ltd, Mumbai, India.

Precision: 0.1 mg.

Immersion liquid: distilled water.

d. XRD:

Instrument: Rigaku Miniflex 600 X-ray Diffractometer, Rigaku, Tokyo, Japan, Cu-Kα target.

Range:2θ = 5^°^ to 80^°^. 

Scanning rate: 1 °/min.

e. FTIR spectroscopy:

Instrument: Shimadzu FTIR 8300 spectrometer, Shimadzu Scientific Instruments, Kyoto, Japan and KBr pellet technique.

Range: 400–4000 cm^−1^.

Resolution: 2 cm^−1^.

f. UV/Vis/NIR absorption spectroscopy:

Instrument: Perkin Elmer Lambda 750s UV-Vis-NIR spectrophotometer, PerkinElmer, Inc., Waltham, MA, USA.

Range: 250–2000 nm.

Resolution: 1 nm.

g. ESR:

Instrument: JEOL-JES FA200 CW ESR Spectrometer, JEOL Ltd., Tokyo, Japan.

Frequency: X-band (9.44 GHz), modulation of 100 kHz.

Measurement temperature: room temperature.

Field center: 250 mT, modulation field width 0.35 mT

Time constant: 0.03 s, sweep time 2 min.

Power: 0.99800 mW.

## 3. Results and Discussion

### 3.1. Thermoluminescence (TL)

**Figure 1:**
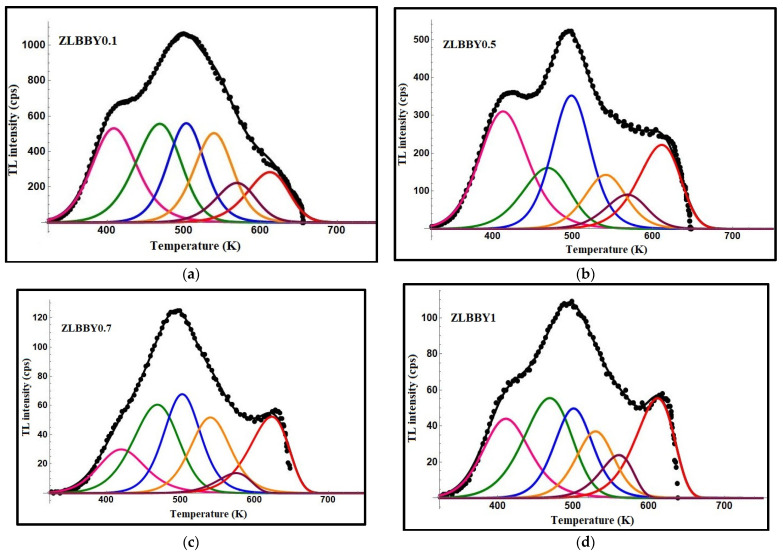
TL graphs of (**a**) ZLBBY0.1 (**b**) ZLBBY0.5 (**c**) ZLBBY0.7 (**d**) ZLBBY1 glasses.

Previously [13], to ascertain the optimum Yb^3+^ concentration, all the glasses were exposed to 3 kGy as a trial dose and their TL graphs were overlaid. The TL graphs of the glasses are given separately here in Figure 1a–d. The ZLBBY glasses exhibited a main broad dosimetric peak positioned about the peak temperature, $T_{m}$ = 500 K. In the temperature span of 325 to 660 K, the 0.1 mol% Yb^3+^-containing glass showed the highest integrated TL intensity among all the glasses. Concentration quenching was, however, noticed for >0.1 mol% Yb^3+^ content gradually until 1 mol%. The integrated TL intensity of the 1 mol% glass was reduced by nearly 9.4 times in comparison with that of the 0.1 mol% Yb^3+^-doped glass.

The TL graphs of the ZLBBY glasses were subjected to computerized glow curve deconvolution (CGCD) by applying Kitis general order relation (Equation (1)), famously used in TL studies [28].

(1) $$I\;\left(T\right)\;=\;I_{m}b^{\left(\frac{b}{b-1}\right)}exp\left(\frac{E}{k_{B}T}\frac{T-T_{m}}{T_{m}}\right){\left[\left(b-1\right)\left(1-\frac{2k_{B}T}{E}\right)\frac{T^{2}}{T_{m}^{2}}\exp\left(\frac{E}{k_{B}T}\frac{T-T_{m}}{T_{m}}\right)+1\;\left(b-1\right)\left(\frac{2k_{B}T}{E}\right)\right]}^{-\left(\frac{b}{b-1}\right)}$$

In the above, $I_{m}$ implies the maximum peak intensity corresponding to $T_{m}$, b is the kinetic order, and $k_{B}$ is the Boltzmann constant. The accuracy of the fit between the theoretical and experimental data was maintained by changing the simulation parameters (E, b, $T_{m}$) and the component peak number various times till the smallest figure of merit (FOM) of <5% was reached [13]. The deconvoluted graphs of the ZLBBY glasses with the component peaks are given in Figure 1a–d. All the glasses were fitted with FOM within the acceptable range and contained six component peaks. Each component peak corresponds to a trap center. The parameters related to a trap or the trap parameters viz. activation energy (E), frequency factor (s), and lifetime of an electron in the trap (τ) calculated using the relations in [13] are presented in Table 1. The thermal release of the electrons from their occupied traps depends on the trap parameters and the temperature at which the material is held following its irradiation. The value of E yields the information on the minimum energy required to drain the trap. The determination of the trap parameter τ gives an insight into the period of the electron in the trap. The trap parameter s quantifies the ‘effort to escape’ nature of the electron from the trap. For each TL glow curve, the activation energy was greater for the deconvoluted peaks at relatively higher temperatures. This evidences that the high temperature peaks are due to the deeper traps formed inside the material, whereas the lower temperature peaks correspond to the shallow traps needing lower energy for de-trapping [29]. Among the prepared glasses, the ZLBBY0.1 glass with an FOM value of 3.02% showed the highest range of trap activation energies and lifetimes.

Table 2 presents the trap parameters of the ZLBBY glasses computed by Chen’s peak shape method [30]. The geometrical factor (${µ}_{g}$) ranged from 0.42 to 0.53. The trap density or the density of the trapped charges ($n_{0}$ cm^−3^) in each glass was determined by the relation in [20] as below:

(2) $$n_{0}\;=\;\frac{\omega I_{m}}{\beta \left\{2.52+10.2\left(\mu_{g}-0.42\right)\right\}}$$

where ω is the total half-width of the peak [28] and β is the heating rate. The ZLBBY0.1 glass contained traps with the highest density ranging from 1.47 × 10^3^ to 4.57 × 10^3^ cm^−3^.

### 3.2. X-ray Diffraction (XRD)

**Figure 2:**
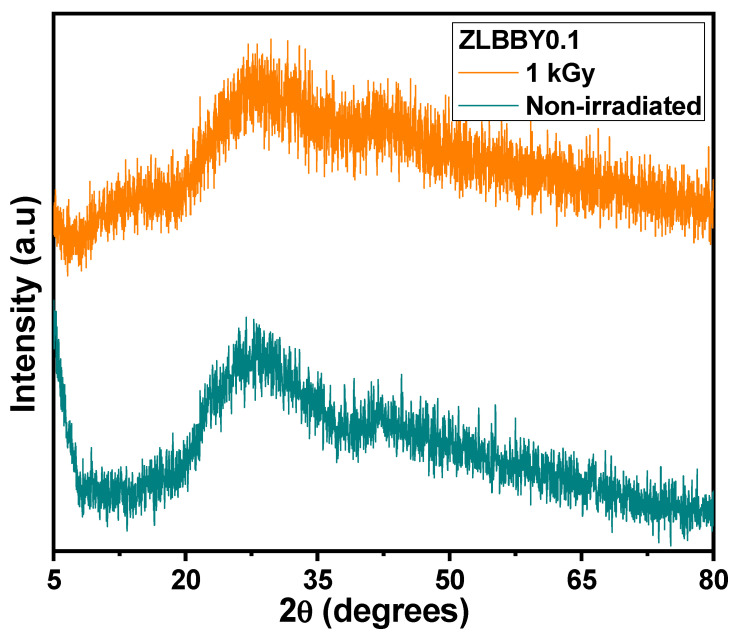
Stacked XRD profiles of the non-irradiated and the 1 kGy γ-irradiated ZLBBY0.1 glasses.

The stacked X-ray diffractograms of the non-irradiated and the optimum dose 1 kGy γ-irradiated ZLBBY0.1 glasses are shown in Figure 2. A broad diffused hump spread over 2θ = 20–70° was exhibited by both the samples. No specific crystalline peaks were detected. This confirmed their amorphous nature.

### 3.3. Density

The density of the ZLBBY0.1 glass before irradiation was obtained from a density balance in our previous work [13] as 3.0161 g/cm^3^. In this work, the density of the ZLBBY0.1 glass after 1 kGy γ-irradiation determined by the same experimental procedure was increased to 3.0600 g/cm^3^. Likewise, N.A. El-Alaily, et al. [7] noticed an increase in density in Li_2_O-B_2_O_3_-based glasses after γ-irradiation. As ionization or atomic displacements due to irradiation may generate electronic defects and bond breakages, the glass structure may relax, and some of the ions may occupy the interstices. The bond angles may become smaller. This may lead the network towards volume compaction, thereby increasing the density [7].

### 3.4. Fourier Transform Infrared (FTIR) Spectroscopy

**Figure 3:**
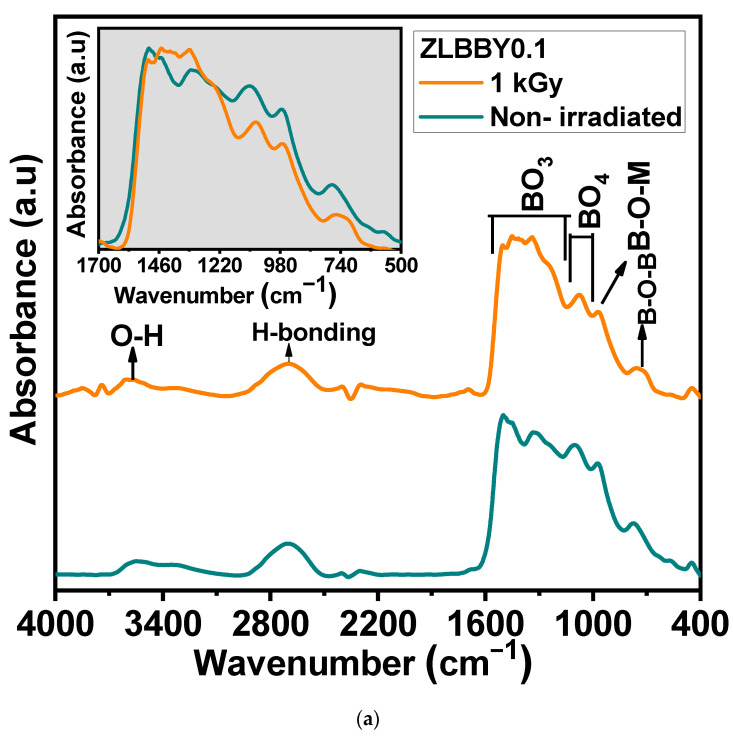

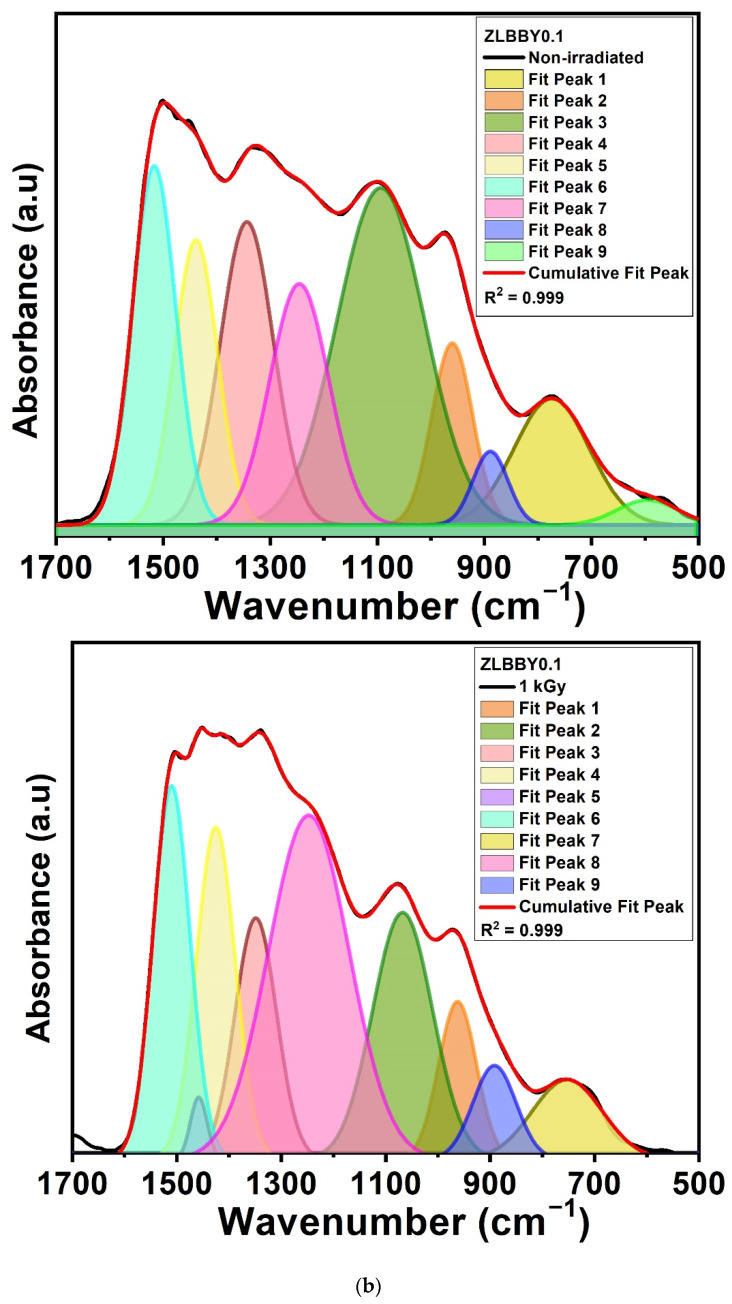
(**a**) Stacked FTIR profiles. The inset represents the overlaid FTIR profiles of the non-irradiated and the 1 kGy γ−irradiated ZLBBY0.1 glasses between 500 and 1700 cm^−1^. (**b**) Deconvoluted FTIR spectra of the non-irradiated and the 1 kGy γ −irradiated ZLBBY0.1 glasses between 500 and 1700 cm^−1^.

The borate glass structure comprises [BO_4_] and [BO_3_] units, which mainly form its skeleton [31]. The FTIR spectrum of a borate glass is generally distributed into three principal regions [7]—(1) 600–800 cm^−1^ (bending vibrations of different borate arrangements), (2) 800–1200 cm^−1^ (vibrations corresponding to [BO_4_] units), and (3) 1200–1500 cm^−1^ (vibrations corresponding to [BO_3_] units). The FTIR spectroscopy of the optimum ZLBBY0.1 glass before and after 1 kGy γ-irradiation was recorded to examine the structural modifications. The stacked FTIR spectra of the two samples are displayed in Figure 3a. The inset of Figure 3a contains the overlaid FTIR profile in the span of 500–1700 cm^−1^ of the ZLBBY0.1 glass before and after 1 kGy γ-irradiation. This wavenumber range was deconvoluted (Figure 3b) using Gaussian function with *R*^2^ ≈ 0.999 into component peaks, which were assigned to the vibrations equivalent to borate units following the literature [7,9]. The peak assignments corresponded to B-O-M (M being any metal ion in the composition), B-O-B, B-O-H, -O_3_-B-F, [BF_3_], [BO_3_], and [BO_4_] units’ vibrations, as presented in Table 3, with their respective peak centers. The characteristic bands of the -H stretching in OH^-^ groups, free OH^-^ groups stretching, and B-O-H vibrations were also noticed before and after irradiation. After irradiation, no considerable modifications in the current positions of the peaks were perceived. However, the structural rearrangement may be reasoned for the appearance and disappearance of some bonds after γ-irradiation. The fractions of 3-coordinated ($N_{3}$) and 4-coordinated boron units ($N_{4}$) in the glasses were estimated using the equations below [32]:

(3) $$N_{3}\;=\;\frac{Area\left(BO_{3}\right)}{Area\left(BO_{3}\right)+Area\left(BO_{4}\right)}$$

(4) $$N_{4}\;=\;\frac{Area\left(BO_{4}\right)}{Area\left(BO_{3}\right)+Area\left(BO_{4}\right)}$$

After 1 kGy γ-irradiation, the value of $N_{4}$ decreased from 0.36 to 0.23 and $N_{3}$ increased from 0.64 to 0.77. The same was analogous to the drastic reduction in the intensity and area under BO_4_ units (800–1200 cm^−1^) and rise under BO_3_ units (1200–1500 cm^−1^) in the 1 kGy γ-irradiated glass when compared with the non-irradiated one, as seen from the Figure 3a inset. The [BO_4_] units may have converted into [BO_3_] units (evident from the rise in $N_{3}$) and non-bridging oxygens (NBOs), owing to the radiolysis of B-O bonds.

### 3.5. Optical Absorption Spectroscopy

**Figure 4:**
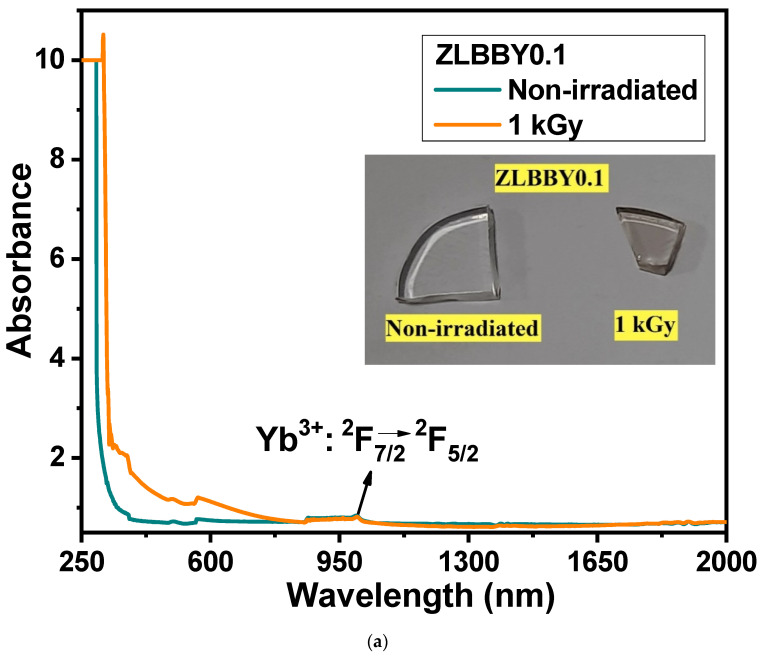

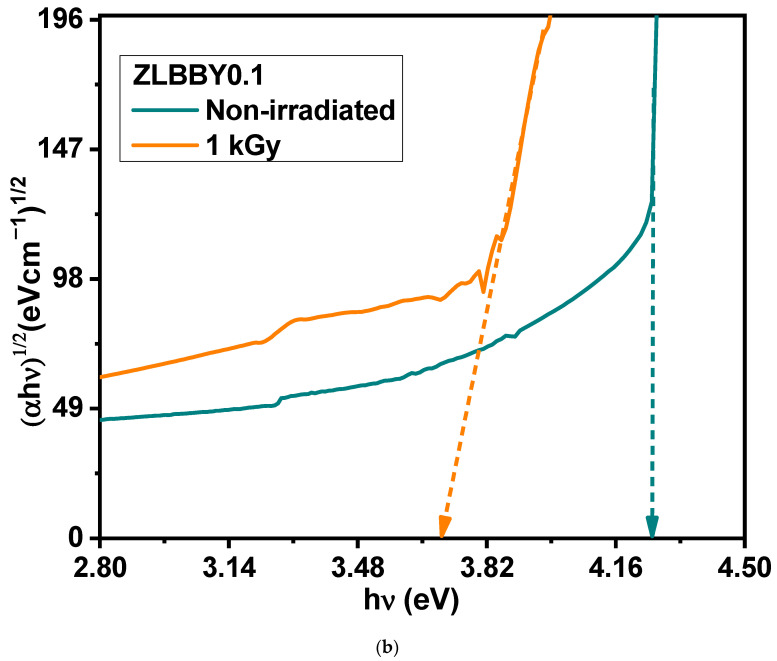
(**a**) Overlaid optical absorption spectra of the non-irradiated and the 1 kGy γ-irradiated ZLBBY0.1 glasses. The inset contains the photograph of the glasses. (**b**) Tauc’s plots of the non-irradiated and the 1 kGy γ -irradiated ZLBBY0.1 glasses.

The UV/Vis/NIR optical absorption spectra of the non-irradiated and the 1 kGy γ-irradiated ZLBBY0.1 glasses are presented in Figure 4a. The inset of Figure 4a contains the photograph of the glasses. The color of the glass turned from colorless to light brown after 1 kGy γ-irradiation. The wavelength range between 250 and 700 nm seemed to have been more affected as the absorption intensity here was significantly enhanced after irradiation. This primarily occurs because of the breakage of bonds, leading to the displacement of ions after γ-rays have impinged on the glass. As a result, the non-bridging oxygen hole centers, boron-oxygen hole centers, boron electron centers, and metal ion interstitials are the commonly found potential trap centers in borate glasses. These trap centers, or ‘color centers’ as they are commonly called, may absorb light of specific wavelengths causing the blackening of the glass after irradiation [11,19]. The absorption spectrum after irradiation contains the superposition of many absorption bands conforming to the various color centers. The band around 215 nm referred to the *E’* center in γ-irradiated silica glasses [11], while the band around 580 nm in γ-irradiated strontium borate glass referred to the positive hole center [9]. In most cases, the bands produced in the UV region after irradiation are connected to the electron centers, while the visible induced absorption is assigned to the hole centers [9,19]. In Figure 4a, although the area under the 1 kGy γ-irradiated absorption curve in the UV/Vis region showed an increment, it is difficult to exactly differentiate and designate the absorption centers. As the defect states are within the forbidden gap, owing to the spectral transitions from the valence band to these defect states and from the defect states to the conduction band, the total absorption of the glass may increase [21]. Another observation was the red-shifting of the cut-off wavelength from 291 to 335 nm after γ-irradiation. This may be attributed to the transfer of some B atoms from [BO_4_] to [BO_3_] configuration [8]. This could have increased the NBOs [21], as confirmed through FTIR spectroscopy, which increases the number of electrons, reducing the energy essential for the transition across the bandgap [21]. The typical broad absorption peak of the Yb^3+^ ion conforming to its only transition ^2^F_7/2_→^2^F_5/2_ was spotted between 900 and 1050 nm. The γ-radiation had little influence on the intensity of this transition and there was no optical degradation.

The optical bandgap for indirect allowed transition $\left(E_{g}\right)$ was evaluated using the Mott and Davis relation [33]. Using the Tauc’s plot (shown in Figure 4b), the bandgaps were determined to be 4.25 eV for the non-irradiated and 3.70 eV for the 1 kGy γ-irradiated ZLBBY0.1 glasses. As expected, the decrease in $E_{g}$ confirmed the formation of color centers introducing new energy levels between the valence and the conduction bands. The reduction in $E_{g}$ is also attributed to the rise in NBOs as they bind the excited electrons less tightly than BOs [8].

Urbach energies (ΔE) [34] of the ZLBBY0.1 glass pre- and post-irradiation were determined to be 0.14 eV and 0.37 eV, respectively. An increase in ΔE was noticed. The growth in the NBOs after irradiation interpreted from the FTIR spectroscopy seconds this notion. As the magnitude of the negative charges on the NBOs is greater than that on the BOs, the band tailing may be so distinct to decrease the optical bandgap and increase Urbach energy [8]. The rise in Urbach energy also implies the increase in the structural defects and disorder resulting from the breaking of the bonds upon irradiation [8].

### 3.6. Electron Spin Resonance (ESR)

**Figure 5:**
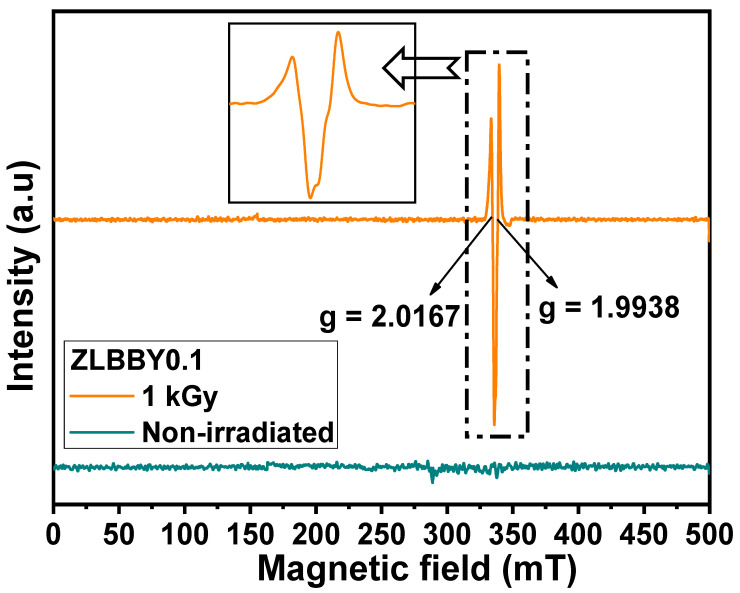
Stacked ESR profile of the non-irradiated and the 1 kGy γ-irradiated ZLBBY0.1 glasses.

ESR is a popular technique to further examine the generation of various color centers or paramagnetic defects in a material. The characteristic ‘g-value’ in the ESR spectrum helps in identifying these centers. Generally, in glass, these centers are associated with the electrons/holes trapped at diverse locations in the structure. The ESR spectra in the double derivative mode of the non-irradiated and 1 kGy γ-irradiated 0.1 mol% Yb_2_O_3_-doped glasses are stacked in Figure 5. No paramagnetic signals were detected in the ESR profile of the non-irradiated ZLBBY0.1 glass. It is interesting to observe that the 1 kGy γ-irradiated ZLBBY0.1 glass exhibited two intense signals (Figure 5 and inset) at g = 2.0167 and g = 1.9938. As these signals were absent in the non-irradiated glass, it implies that the structural changes induced as a result of irradiation have created paramagnetic centers. The B-O bond is more likely to be affected by radiation [7]. Like the g = 2.0167 center in the 1 kGy ZLBBY0.1 glass, a signal at g = 2.0128 was noticed in a commercial window glass irradiated with 6 kGy γ-dose [35], and a signal at g = 2.0130 in a bio-glass irradiated with 100 Gy γ-dose [36], both of which were linked to the non-bridging oxygen hole center (NBOHC). Similarly, in another work [17], the effective resonance signal greater than the g-value of a free electron (g = 2.0023) was credited to the trapped holes on the NBOs and [BO_4_] units. The NBOHC, which is in general a hole trapped on a non-bridging oxygen (≡B−O), in the case of borate glasses may be formed by O–H bond radiolysis, as below [12]:

(5) $$\equiv B-OH\overset{irradiation}{\to }\equiv B-O\cdot +H{}^{\circ}$$

Here, the notation ‘·’ means an unpaired localized electron [17]. The H° appearing in the above equation is observed when irradiation and ESR measurement are performed together at temperatures of ≤100 K and at temperatures of ≥120 K; the species becomes mobile and vanishes [2].

The 1 kGy γ-irradiated ZLBBY0.1 glass also exhibited a center with g-value < 2 at g = 1.9938. An intense signal around g = 2.000 in silica glasses was linked to the *E’* center [11]. In borate glasses, the *E’*-center or the Boron *E’*-center is a typical paramagnetic defect with unpaired electron on a boron bonded to three oxygens (≡B) [2]. The NBOHC and the *E’*-centers may develop from the precursors in the glass composition after irradiation [12]. Usually, the NBOHC and *E’*-center may be produced together out of the strained bond cleavage of B-O as below [12]:

(6) $$\equiv B-O-B\equiv \;\overset{irradiation}{\to }\;\equiv B\cdot \;\cdot O-B\equiv$$

The trapped electron centers and trapped hole centers are always generated in pairs; hence the *E’*-center coexists with the NBOHC in irradiated glasses [11,12]. However, owing to the fast spin-lattice relaxation time of the rare-earth ions, the characteristic of the ytterbium ions was not observed at room temperature as liquid helium temperature is needed to induce an ESR signal. As a future scope of the work, ESR at different temperatures can be taken up to establish a relation between defect centers elucidated from the ESR and TL studies.

## 4. Conclusions

The structural and optical modifications after γ-irradiation on the BaO-ZnO-LiF-B_2_O_3_*-*Yb_2_O_3_ glass system were studied. The optimum glass sample (10BaO-20ZnO-20LiF-49.9B_2_O_3_-0.1Yb_2_O_3_) containing the relatively higher trap density and deeper traps was analyzed for the radiation-induced changes through XRD, density, FTIR, optical absorption, and ESR techniques. The XRD of the glass confirmed its amorphous nature before and after irradiation. The glass density was increased after irradiation. The transformation of [BO_4_] units into [BO_3_] units and non-bridging oxygens was elucidated from the FTIR analysis. The color of the glass darkened after irradiation. This was correlated to the formation of color centers, evident from the increase in the UV/Vis optical absorption intensity, reduction in optical bandgap, and increase in Urbach energy. The ESR profile of the irradiated glass exhibited two signals at g = 2.0167 and g = 1.9938, which corresponded to the non-bridging oxygen hole center and Boron *E’*-center, respectively. The work can be continued in future by considering the ESR at different temperatures to establish a relation between defect centers elucidated from the ESR and TL studies.

## Figures and Tables

**Table 1:** Trap parameters of the ZLBBY glasses computed by the CGCD technique.

Sample	Peak No.	$T_{m}$ (K)	b	E (eV)	Frequency Factor s (s^−1^)	Lifetime τ (Years)	FOM (%)
ZLBBY0.1 [13]	1	408	1.79	0.63	7.23 × 10^6^	8.40 × 10^−4^	3.02
	2	468	1.29	0.69	2.82 × 10^6^	6.53 × 10^−3^	
	3	503	1.70	1.16	6.39 × 10^10^	55.50	
	4	539	1.59	1.23	4.49 × 10^10^	872	
	5	569	1.39	1.24	1.25 × 10^10^	3.11 × 10^3^	
	6	612	1.22	1.29	4.95 × 10^9^	4.26 × 10^4^	
ZLBBY0.5	1	412	1.79	0.62	4.41 × 10^6^	9.34 × 10^−4^	
	2	468	1.29	0.69	2.82 × 10^6^	6.53 × 10^−3^	
	3	498	1.70	1.14	5.26 × 10^10^	31.10	3.59
	4	541	1.61	1.21	2.58 × 10^10^	735	
	5	568	1.39	1.24	1.31 × 10^10^	2.97 × 10^3^	
	6	611	1.12	1.27	3.50 × 10^9^	2.46 × 10^4^	
ZLBBY0.7	1	419	1.79	0.61	2.37 × 10^6^	1.18 × 10^−3^	
	2	468	1.29	0.67	1.66 × 10^6^	5.10 × 10^−3^	
	3	500	1.70	1.11	2.27 × 10^10^	22.52	3.18
	4	540	1.71	1.19	1.72 × 10^10^	682	
	5	575	1.02	1.24	9.64 × 10^9^	2.5 × 10^3^	
	6	623	1.01	1.26	1.76 × 10^9^	2.95 × 10^4^	
ZLBBY1	1	410	1.80	0.60	2.65 × 10^6^	7.51 × 10^−4^	
	2	468	1.20	0.62	4.48 × 10^5^	2.42 × 10^−3^	
	3	500	1.70	1.09	1.40 × 10^10^	16.85	4.00
	4	529	1.55	1.17	1.96 × 10^10^	178	
	5	560	1.02	1.23	1.60 × 10^10^	1.02 × 10^3^	
	6	611	1.01	1.25	2.38 × 10^9^	1.48 × 10^4^	

**Table 2:** Trap parameters of the ZLBBY glasses computed by Chen’s peak shape technique.

Sample	Peak No.	$T_{m}$ (K)	${µ}_{g}$	Activation Energy (eV)				Frequency Factor s (s^−1^)	Lifetime τ (Years)	$n_{0}$ ×10^3^ (cm^−3^)
				$E_{\tau }$	$E_{\delta }$	$E_{\omega }$	E			
ZLBBY0.1 [13]	1	408	0.48	0.56	0.62	0.59	0.59	2.11 × 10^6^	1.28 × 10^−2^	4.16
	2	468	0.46	0.69	0.76	0.73	0.73	7.50 × 10^6^	8.21 × 10^−1^	4.57
	3	503	0.49	1.13	1.16	1.15	1.15	4.92 × 10^10^	1.47 × 10^3^	3.48
	4	539	0.47	1.18	1.23	1.21	1.21	2.78 × 10^10^	2.66 × 10^4^	3.73
	5	569	0.48	1.20	1.25	1.23	1.23	9.62 × 10^9^	1.67 × 10^5^	1.47
	6	612	0.45	1.30	1.35	1.33	1.33	1.03 × 10^10^	7.53 × 10^6^	2.16
ZLBBY0.5	1	412	0.52	0.61	0.66	0.64	0.64	7.86 × 10^6^	2.39 × 10^−2^	2.24
	2	468	0.46	0.64	0.72	0.68	0.68	2.00 × 10^6^	4.43 × 10^−1^	1.39
	3	498	0.50	1.08	1.11	1.10	1.10	1.95 × 10^10^	5.35 × 10^2^	2.21
	4	541	0.48	1.15	1.20	1.18	1.18	1.28 × 10^10^	1.80 × 10^4^	0.97
	5	568	0.49	1.17	1.22	1.20	1.20	5.32 × 10^9^	9.44 × 10^4^	0.64
	6	611	0.46	1.22	1.28	1.26	1.25	2.19 × 10^9^	1.59 × 10^6^	1.74
ZLBBY0.7	1	419	0.51	0.61	0.66	0.64	0.63	4.18 × 10^6^	3.05 × 10^−2^	0.24
	2	468	0.45	0.65	0.72	0.69	0.69	2.61 × 10^6^	5.01 × 10^−1^	0.52
	3	500	0.49	1.03	1.08	1.06	1.06	6.60 × 10^9^	3.34 × 10^2^	0.45
	4	540	0.48	1.11	1.16	1.14	1.13	4.38 × 10^9^	7.60 × 10^3^	0.35
	5	575	0.44	1.23	1.26	1.25	1.25	1.10 × 10^10^	3.16 × 10^5^	0.09
	6	623	0.42	1.44	1.43	1.45	1.44	5.38 × 10^10^	1.02 × 10^8^	0.38
ZLBBY1	1	410	0.53	0.59	0.63	0.62	0.61	3.51 × 10^6^	1.67 × 10^−2^	0.34
	2	468	0.47	0.62	0.69	0.66	0.66	1.18 × 10^6^	3.46 × 10^−1^	0.49
	3	500	0.50	1.09	1.12	1.11	1.11	2.22 × 10^10^	6.91 × 10^2^	0.30
	4	529	0.46	1.10	1.15	1.13	1.13	7.59 × 10^9^	4.39 × 10^3^	0.22
	5	560	0.43	1.23	1.24	1.24	1.24	1.85 × 10^10^	1.28 × 10^5^	0.16
	6	611	0.45	1.24	1.29	1.27	1.27	3.27 × 10^9^	2.32 × 10^6^	0.43

**Table 3:** FTIR peak assignments of the ZLBBY0.1 glass.

Assignment	Peak Center (cm^−1^)	
	Non-Irradiated	1 kGy
Zn^2+^, Li^+^, Ba^2+^, or any metal cations	595	-
B-O-B linkages bending vibrations	774	752
[BO_4_] units stretching vibrations	888	891
Stretching of B–O–M	960	962
-O_3_-B-F vibrations	1093	1067
B-O^-^ stretching vibrations in [BO_3_] units	1245	1247
B–O stretching vibrations in [BO_3_] units of metaborate, pyroborate, and orthoborate groups	1343	1348
Stretching of NBOs in [BO_3_] units	1438	1425
B-F vibrations in [BF_3_] units	-	1457
B-O anti-symmetric stretching vibrations in [BO_3_] units	1516	1509
-H stretching in OH^-^ groups	2685	2698
OH^-^ groups and B-O-H vibrations	3357 3561	3592 3740 3844

## Data Availability

The data presented in this study are available on request from the corresponding author.
